# Association of erectile dysfunction and peripheral arterial disease in NHANES 2001-2004: a cross-sectional study

**DOI:** 10.3389/fendo.2024.1439609

**Published:** 2024-11-06

**Authors:** Ganggang Wang, Caifang Ni

**Affiliations:** ^1^ Department of Interventional and Vascular Surgery, The Affiliated Changzhou Second People's Hospital of Nanjing Medical University, Changzhou, China; ^2^ Department of Interventional Radiology, First Affiliated Hospital of Soochow University, Suzhou, Jiangsu, China

**Keywords:** erectile dysfunction, peripheral arterial disease, ankle-brachial index, cross-sectional study, NHANES

## Abstract

**Objective:**

To evaluate the association between Erectile dysfunction (ED) and peripheral arterial disease (PAD) in adult American males using a large database.

**Methods:**

The relationship between ED and PAD prevalence among participants in the 2001-2004 National Health and Nutrition Examination Survey (NHANES) database was assessed using a series of statistical analyses. ED was evaluated based on a single-item measure of self-reported erection problems from the Massachusetts Male Aging Study. PAD was defined as ankle-brachial index (ABI) < 0.9 in at least one leg. Multifactorial logistic regression models were used to investigate the association between ED and PAD.

**Results:**

A total of 2394 participants were enrolled, of whom 905 individuals (37.8%) were diagnosed with ED. After adjusting for confounding variables, the association between ED and PAD remained positive, with an odds ratio of 2.05 (95% confidence interval 1.24-3.39). Subgroup analysis revealed that the relationship between ED and PAD was significant in patients aged >50 years old, without hypertension, without diabetes, without cardiovascular disease, without high cholesterol, former smokers, low physical activity levels, and a body mass index of 25-30 (P < 0.05). In addition, all subgroups analyzed were evaluated for any potential interaction, and no statistically significant association was discovered.

**Conclusions:**

In a sample of US adults aged ≥40, this cross-sectional study found that ED is related to a higher occurrence of PAD. ED may be an independent predictor of PAD, and thus it should be considered in the treatment of patients with ED.

## 1 Introduction

Erectile dysfunction (ED) is estimated to affect approximately 152 million males globally, with the prevalence projected to rise to 322 million cases by the year 2025 (1). Increasing evidence suggests a strong link between erectile dysfunction and atherosclerosis (2, 3). Several meta-analyses have shown that erectile dysfunction significantly increases the likelihood of experiencing a stroke, coronary heart disease (CVD), cardiovascular disease, and death from any cause (4, 5).

Peripheral arterial disease (PAD) is a significant cardiovascular condition caused by atherosclerosis, mainly affecting the arteries in the legs (6). The global prevalence of PAD continues to ascend annually, impacting over 200 million individuals, particularly middle-aged and elderly individuals (7). Nevertheless, PAD is often undiagnosed due to a lack of PAD-related knowledge and awareness and a high number of asymptomatic cases (8). Despite the established link between ED and atherosclerosis, the connection between ED and PAD is poorly understood. Reports on the association between ED and PAD are scanty, with only a few studies examining specific populations, such as diabetic individuals or those at elevated risk for CVD (9–11). Therefore, whether ED is a predictor of PAD or whether it can be utilized to identify individuals who would benefit from PAD screening remains elusive.

The study sought to investigate the link between ED and PAD by analyzing the National Health and Nutrition Examination Survey (NHANES) data collected between 2001 and 2004.

## 2 Materials and methods

### 2.1 Study population

The study utilized data from the NHANES database, a major program of the National Center for Health Statistics (NCHS), which is part of the Centers for Disease Control and Prevention (CDC). NHANES applies a sophisticated, multistage, probability sampling design to assess the dietary intake, health, and nutritional status of noninstitutionalized adults and children in the United States (12). Standardized interviews, physical exams, and laboratory tests are conducted to gain an understanding of various population demographics. NHANES data have been available for research since 1999 and are released biennially. Data from the 2001-2002 and 2003-2004 NHANES cycles were used in the present study. More details are available on the NHANES website (https://www.cdc.gov/nchs/nhanes/).

Only the data sets from the 2001-2002 and 2003-2004 NHANES cycles were chosen for cross-sectional analysis due to the lack of ED and ankle-brachial index (ABI) values in other NHANES cycles. Between 2001 and 2004, 21161 people participated in NHANES. Participants were excluded based on the following criteria: being female (n = 10860); missing ED data (n = 6185); previously diagnosed with prostate cancer (n = 36); missing ABI data (n = 1638); having an ABI ≥ 1.4 (n = 48). A total of 2394 cases were included in the final analysis, encompassing 905 individuals with ED and 1489 controls.

### 2.2 Data collection and definition

#### 2.2.1 Assessment of ED

Private interviews were carried out at the MEC using audio computer-assisted self-interview (ACASI) methodology. Evaluating ED involved answering a specific question from the Massachusetts Male Aging Study: “How would you describe your ability to get and keep an erection adequate for satisfactory intercourse?” Response options included “always or almost always able”, “usually able”, “sometimes able”, and “never able”. Participants were categorized as having ED if they reported being ‘sometimes able’ or ‘never able’, while those who reported being ‘always or almost always able’ or ‘usually able’ were classified as not having ED (13).

#### 2.2.2 Assessment of PAD

PAD was defined as ABI < 0.9 in one leg (14). Systolic blood pressure (SBP) was measured on the brachial artery of one arm, with a preference for the unaffected arm if there were doubts about the measurement on the right arm. Furthermore, the SBP in both ankles was assessed utilizing the posterior tibial artery. The ABI was calculated as the systolic ankle pressure divided by the systolic arm pressure on both sides.

### 2.3 Covariates of interest

Variables were chosen according to established confounding factors in prior research and medical practices. Various variables were analyzed, including age, ethnicity, level of education, marital status, family income, hypertension, diabetes, CVD, high cholesterol, smoking habits, alcohol consumption, physical activity, and body mass index (BMI) (15).

Individuals were categorized based on ethnicity (Mexican American, Other Hispanic, Non-Hispanic White, Non-Hispanic Black, and other ethnicities, including American Indian or Alaska Native, Native Hawaiian, other Pacific Islander, and individuals of mixed race), education levels (less than high school, high school, and more than high school), marital status (married/living with partners, widowed/divorced/separated, and never married), household poverty-to-income ratio (PIR) (low (≤1.3), medium (1.3-3.5), or high (≥3.5)), and blood pressure (SBP ≥ 140 mmHg or diastolic blood pressure (DBP) ≥ 90 mmHg). Diabetes was characterized by self-reported diabetes or glycated hemoglobin level of ≥6.5% (16). CVD was defined as individuals who were previously diagnosed with congestive heart failure, coronary artery disease, angina, or a heart attack. Those diagnosed with high blood cholesterol levels were given medication for hypercholesterolemia; individuals with a total cholesterol reading of ≥240 mg/dL were categorized as having elevated cholesterol. Individuals who had previously smoked a minimum of 100 cigarettes and were presently smoking during the survey were categorized as current smokers. Those who had previously smoked at least 100 cigarettes in their lifetime and were not currently smoking during the survey were classified as former smokers. Meanwhile, males who had previously smoked less than 100 cigarettes in their lifetime were categorized as nonsmokers. Participants who had consumed at least 12 alcoholic beverages in their lifetime or within a year and had consumed alcohol within the previous year were classified as current drinkers. Participants were categorized into three groups based on their self-reported leisure-time physical activity levels: inactive, moderate, and vigorous. Additionally, the body mass index (BMI) was categorized into three groups: underweight/normal weight (<25.0 kg/m^2^), overweight (25.0-29.9 kg/m^2^), and obese (≥30.0 kg/m^2^).

### 2.4 Statistical analysis

Continuous variables were presented as weighted means with standard deviations and analyzed using either the independent samples T-test or the Mann-Whitney test. Categorical variables were expressed as weighted percentages along with 95% confidence intervals (95% CI) and assessed using the χ^2^ test. Multivariate logistic regression was performed to investigate the relationship between ED and PAD. Three distinct models were employed: Model 1 without modifications, Model 2 with adjustments for age, race, and education level, and Model 3 with further adjustments for marital status, PIR, hypertension, diabetes, CVD, high cholesterol, smoking, alcohol consumption, physical activity, and BMI.

Subgroup analyses and interaction tests were also conducted. All data were analyzed using R software (The R Foundation, http://www.R-project.org) and Empower Stats (X&Y Solutions, Inc., Boston, MA, http://www.empowerstats.com). The sample weights were created to account for the complex survey design. Two-sided P < 0.05 was considered statistically significant.

## 3 Results

### 3.1 Characteristics of the participants

**Figure 1:**
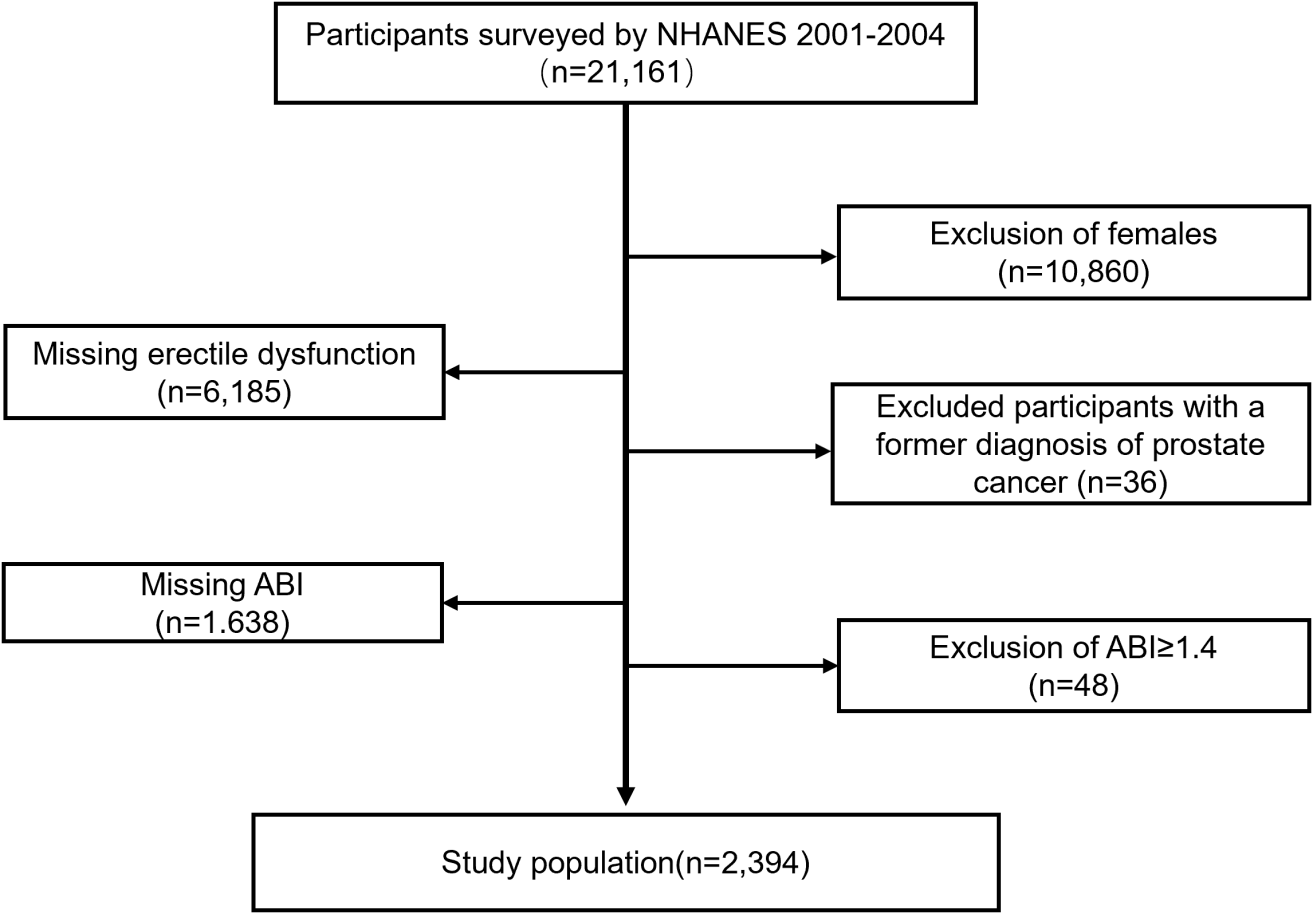
Flow chart of the screening process for the selection of study participants. ABI, ankle-brachial index.

A total of 21161 individuals participated in 2001-2004 NHANES. After a meticulous screening procedure, 2394 participants met the eligibility criteria, of whom 905 individuals (37.8%) were diagnosed with ED. The specific selection process of study participants is depicted in **Figure 1**. The baseline characteristics of the participants and a weighted examination of the characteristics of the study group are displayed in **Table 1**. The prevalence of PAD in men with ED was >4 times higher than that in men without ED (11.4% vs. 2.6%, P < 0.001). Compared with the non-ED group, individuals in the ED cohort were typically older, with lower education levels, PIR, and physical activity levels, and a higher incidence of hypertension, diabetes, CVD, high cholesterol, and a history of smoking, as well as a higher BMI.

**Table 1:** General characteristics of included participants (n = 2394) by the presence or absence of erectile dysfunction in the NHANES 2001-2004.

Characters	Total	NO (N=1489)	YES (N=905)	*P*-value
Age(years)	54.8 ± 11.4	51.3 ± 9.3	64.1 ± 11.4	<0.001
≤50	43.5 (40.3-46.7)	55.1 (51.4-58.8)	12.6 (9.5-16.4)	
>50	56.5 (53.3-59.7)	44.9 (41.2-48.6)	87.4 (83.6-90.5)	
Race/ethnicity				0.469
Mexican American	4.6 (3.2-6.7)	4.8 (3.5-6.5)	4.3 (2.2-8.1)	
Other Hispanic	3.7 (2.1-6.4)	3.2 (1.9-5.5)	4.8 (2.1-10.4)	
Non-Hispanic White	80.1 (76.1-83.7)	80.3 (76.2-83.8)	79.8 (74.3-84.4)	
Non-Hispanic Black	8.7 (7-10.9)	8.9 (7.2-11)	8.3 (6.3-10.9)	<0.001
Other Race^a^	2.8 (2-4)	2.8 (1.7-4.6)	2.8 (1.7-4.5)	
Education level				0.021
Less than high school	15.5 (13.5-17.7)	11.6 (9.8-13.7)	25.9 (21.6-30.6)	
High school	25.7 (23.8-27.7)	26.9 (24.4-29.6)	22.3 (18.9-26.2)	
More than high school	58.9 (55.5-62.1)	61.5 (58.2-64.7)	51.8 (47.5-56.2)	
Marital status				<0.001
Married/Living with partners	5.9 (4.5-7.7)	6.7 (5.1-8.8)	3.8 (2.4-6)	
Widowed/Divorced/Separated	79.1 (76.5-81.4)	78.7 (75.6-81.4)	80.1 (76.6-83.2)	
Never married	15 (13.1-17.2)	14.6 (12.1-17.6)	16.1 (13.7-18.8)	
Family poverty ratio				<0.001
<1.3	12.8 (10.8-14.9)	11.4 (9.7-13.5)	16.2 (12.5-20.9)	
1.3-3.5	30.9 (28.7-33.3)	28.1 (25.2-31.2)	38.4 (34.3-42.8)	
≥3.5	51.4 (47.8-54.9)	55.8 (51.9-59.5)	39.8 (34.8-44.9)	
Not recorded	4.9 (3.8-6.4)	4.7 (3.4-6.4)	5.6 (3.7-8.2)	
Hypertension				<0.001
No	64.6 (60.9-68)	71.3 (66.4-75.7)	46.8 (44-49.7)	
Yes	35.4 (32-39.1)	28.7 (24.3-33.6)	53.2 (50.3-56)	
Diabetes				<0.001
No	87.1 (85.5-88.6)	92 (90.3-93.4)	74.2 (70.9-77.2)	
Yes	12.9 (11.4-14.5)	8 (6.6-9.7)	25.8 (22.8-29.1)	
CVD				<0.001
No	86.6 (84.4-88.5)	91.6 (89.7-93.2)	73.2 (69.1-76.9)	
Yes	13.4 (11.5-15.6)	8.4 (6.8-10.3)	26.8 (23.1-30.9)	
High cholesterol				<0.001
No	52.9 (49.8-56)	55.1 (51.8-58.3)	47.1 (42.8-51.5)	
Yes	47.1 (44-50.2)	44.9 (41.7-48.2)	52.9 (48.5-57.2)	
Smoking				<0.001
Never	37.7 (34.6-40.9)	40.7 (36.9-44.6)	29.9 (26.4-33.7)	
Current	23.6 (21.4-25.9)	24.8 (22.5-27.4)	20.3 (16.1-25.3)	
Former	38.7 (36.2-41.2)	34.5 (31.2-37.8)	49.8 (45.2-54.4)	
Alcohol intaking				<0.001
No	25 (21.3-29.1)	22.7 (19-27)	31 (26.3-36.2)	
Yes	75 (70.9-78.7)	77.3 (73-81)	69 (63.8-73.7)	
Physical activity				0.004
Inactive	34.3 (31.6-37.2)	30.8 (27.6-34.3)	43.6 (39.3-48.1)	
Moderate	32.7 (30.6-34.9)	30.8 (28.2-33.6)	37.6 (34-41.4)	
Vigorous	33 (30.3-35.7)	38.3 (35.2-41.5)	18.7 (15.4-22.6)	
BMI				<0.001
<25	22.4 (19.7-25.4)	22.7 (19-26.8)	21.8 (18.9-25.1)	
25-29.99	45 (42.7-47.4)	46.6 (43.7-49.5)	41 (37-45)	
≥30	31.6 (29.2-34)	30.1 (26.9-33.5)	35.5 (31.3-39.9)	
Not recorded	1 (0.6-1.6)	0.7 (0.3-1.4)	1.7 (0.9-3.3)	
PAD				<0.001
No	95 (93.9-96)	97.4 (96.3-98.3)	88.6 (85.7-91)	
Yes	5 (4-6.1)	2.6 (1.7-3.7)	11.4 (9-14.3)	

Values are weighted mean ± SD or weighted % (95% confidence interval). P values are weighted. ^a^Other races include American Indian or Alaska Native, Native Hawaiian or other Pacific Islander, and multiracial persons.

BMI, body mass index; CVD, cardiovascular disease; NHANES, National Health and Nutrition Examination Survey; PAD, peripheral arterial disease.

### 3.2 Association between ED and PAD

Multivariate logistic regression analysis was performed to elucidate the relationship between ED and the prevalence of PAD. Three models were constructed (**Table 2**). The crude model (Model 1) showed an odds ratio (OR) of 4.91 (95% CI 3.08-7.82). After partial adjustment of variables (Model 2), the OR decreased to 2.90 (95% CI 1.81-4.03). Despite full modification in Model 3, including the inclusion of additional variables, the correlation between ED and PAD remained significantly favorable, showing an OR of 2.05 (95% CI 1.24-3.39). Together, these findings suggest a robust association between ED and PAD, persisting even with adjustments for various factors.

**Table 2:** Association between peripheral arterial disease and erectile dysfunction.

Exposure	Model 1 OR (95%CI), *P*	Model 2 OR (95%CI), *P*	Model 3 OR (95%CI), *P*
Erectile Dysfunction			
NO	Reference	Reference	Reference
YES	4.91 (3.08-7.82) <0.001	2.90 (1.81-4.63) <0.001	2.05 (1.24-3.39) 0.007

CI, confidence interval; OR, odds ratio.

Model 1 was unadjusted.

Model 2 was adjusted for age, race, and education level.

Model 3 was adjusted for age, race, education level, marital status, family poverty ratio, hypertension, diabetes, cardiovascular disease (CVD), high cholesterol, smoking, alcohol intaking, physical activity and body mass index (BMI).

### 3.3 Subgroup analysis

Additional analyses were conducted on subgroups based on different variables (**Table 3**). Results showed that ED was positively associated with PAD, with significant relationships found in patients aged >50 years old (OR = 2.32, 95% CI 1.34-4.03), without hypertension (OR = 2.18, 95% CI 1.08-4.40), without diabetes (OR = 2.10, 95% CI 1.14-3.86), without CVD (OR = 2.09, 95% CI 1.14-3.82), without high cholesterol (OR = 2.50, 95% CI 1.32-4.72), former smokers (OR = 3.15, 95% CI 1.36 -7.30), physically inactive individuals (OR = 2.59, 95% CI 1.19-5.61), and those with a BMI of 25-30 (OR = 3.21, 95%CI 1.77-5.84). However, no statistically significant correlation was found after analyzing all subgroups for interaction (all P > 0.05).

**Table 3:** Subgroup analysis for peripheral arterial disease and erectile dysfunction, weighted.

Characteristics	Model 1 OR (95%CI), *P*	Model 2 OR (95%CI), *P*	Model 3 OR (95%CI), *P*	P for interaction
Stratified by Age				0.156
≤50	0.74 (0.07-7.74) 0.795	0.75 (0.08-7.23) 0.799	0.19 (0.04-1.04) 0.055	
>50	3.30 (1.96-5.56) <0.001	3.14 (1.86-5.3) <0.001	2.32 (1.34-4.03) * 0.004	
Stratified by Hypertension				0.268
No	5.35 (2.65-10.8) <0.001	3.29 (2.04-5.28) <0.001	2.18 (1.08-4.40) * 0.015	
Yes	3.17 (1.55-6.45) 0.002	2.32 (1.2-4.49) 0.032	2.05 (0.93-4.54) 0.074	
Stratified by Diabetes				0.71
No	5.09 (2.89-8.95) <0.001	3.01 (1.71-5.29) <0.001	2.10 (1.14-3.86) * 0.019	
Yes	2.48 (1.02-6.03) 0.045	1.6 (0.59-4.32) 0.342	1.26 (0.53-3) 0.597	
Stratified by CVD				0.373
No	4.56 (2.49-8.33) <0.001	2.62 (1.56-4.39) 0.001	2.09 (1.14-3.82) * 0.019	
Yes	2.24 (1.18-4.27) 0.016	2.16 (1.03-4.53) 0.043	2.12 (0.97-4.65) 0.06	
Stratified by High cholesterol				0.133
No	6.21 (3.09-12.48) <0.001	3.51 (2.01-6.14) <0.001	2.50 (1.32-4.72) * 0.006	
Yes	3.93 (2.09-7.37) <0.001	2.39 (1.24-4.62) 0.011	1.71 (0.87-3.37) 0.115	
Stratified by Smoking				0.159
Never	2.82 (1.12-7.14) 0.03	1.62 (0.61-4.31) 0.32	0.86 (0.28-2.63) 0.79	
Current	3.92 (1.84-8.35) 0.001	2.25 (1.21-4.18) 0.012	1.76 (0.95-3.27) 0.07	
Former	6.54 (2.94-14.57) <0.001	4.02 (1.79-9.07) 0.001	3.15 (1.36-7.30) * 0.009	
Stratified by Alcohol intaking				0.837
No	3.47 (1.39-8.69) 0.01	2.95 (1.31-6.63) 0.011	2.43 (1.05-5.61) * 0.038	
Yes	5.58 (3.12-9.99) <0.001	2.99 (1.67-5.35) 0.001	2.15 (1.14-4.04) * 0.019	
Stratified by Physical activity				0.279
Inactive	5.11 (2.46-10.59) <0.001	2.83 (1.35-5.91) 0.007	2.59 (1.19-5.61) * 0.018	
Moderate	2.33 (1.12-4.87) 0.026	1.6 (0.8-3.2) 0.179	1.18 (0.59-2.33) 0.629	
Vigorous	7.71 (2.18-27.3) 0.003	5.46 (1.72-17.3) 0.005	2.01 (0.37-10.86) 0.403	
Stratified by BMI				0.993
<25	4.30 (1.66-11.15) 0.004	2.41 (0.98-5.95) 0.056	1.83 (0.67-5.02) 0.228	
25-29.99	7.47 (4.25-13.13) <0.001	4.3 (2.46-7.53) <0.001	3.21 (1.77-5.84) *<0.001	
≥30	3.22 (1.44-7.18) 0.006	2.09 (0.94-4.63) 0.07	1.29 (0.55-2.99) 0.546	

CI, confidence interval; OR, odds ratio.

Model 1 was unadjusted.

Model 2 was adjusted for age, race, and education level.

Model 3 was adjusted for age, race, education level, marital status, family poverty ratio, hypertension, diabetes, cardiovascular disease (CVD), high cholesterol, smoking, alcohol intaking, physical activity and body mass index (BMI). *‘Inf’ means that values can’t be calculated. *p < .05.

## 4 Discussion

The present cross-sectional study employed data from US adults aged ≥40 years old in 2001-2004 NHANES to investigate the possible link between ED and PAD. The study revealed a significant association between ED and the prevalence of PAD, suggesting that individuals with ED have a higher likelihood of also having PAD. Even after accounting for possible confounding variables, the association remained statistically significant.

Studies on the relationship between ED and PAD have received little attention. A recent meta-analysis found no significant association between ED and PAD. Considerable variability was found in the studies (17). A population-based study involving 614 volunteers found that ED was associated with higher carotid atherosclerosis burdens but not lower extremity atherosclerosis rates. Nevertheless, ABI decreased and the prevalence of ABI < 0.9 increased with an increase in the severity of ED (9). This may be attributed to the limited sample sizes and a less accurate technique for identifying atherosclerosis in the lower limb arteries. Conversely, in the DIVA registry comprising 1366 type 2 diabetic patients, the incidence of abnormal ABI was significantly higher in patients with ED than in those without ED (18). Furthermore, a study of the National Health Insurance Research Database involving 12825 patients who visited the Emergency Department found that men with ED had a 75% increased likelihood of PAD, even after accounting for cardiovascular risk factors and medication usage (19). However, there is a paucity of studies investigating the connection between ED and PAD. The current study utilized extensive and diverse data from 2001-2004 NHANES to investigate the connection between ED and PAD. The findings provide more insights into understanding the association of ED and PAD.

PAD is a chronic obstructive atherosclerotic disease of the arteries from the distal aorta to the foot that disrupts or obstructs blood flow to the feet. Similar to ED, PAD is closely linked to risk factors for atherosclerosis (20). However, PAD is underdiagnosed. Early detection of PAD can result in better cardiovascular results and decrease complications in the lower extremities, including critical limb ischemia and limb loss (21).

Since most risk factors for ED are identical to those for atherosclerotic disease, it can be partially considered a vascular disease (22). In line with previous findings, we discovered that ED was linked to common risk factors for atherosclerosis, including older age, diabetes, high blood pressure, elevated cholesterol levels, and tobacco use. Accumulating evidence indicates that ED is strongly linked to undiagnosed atherosclerotic vascular disease (23–25). According to the artery size hypothesis, all major vascular beds should be affected equally in the presence of atherosclerosis, given its systemic nature. However, symptoms rarely become evident at the same time (26). This implies that the identical typical disease progression could impact smaller blood vessels that provide blood to the penis before affecting larger blood vessels, resulting in the occurrence of ED symptoms before coronary artery disease or PAD symptoms.

The current study demonstrated that the association between ED and the prevalence of PAD was consistent across multiple sub-groups. Age is known to increase the risk of ED and PAD (27, 28). Our subgroup analysis confirmed the association between ED and PAD in older adults. Our subgroup analysis revealed some unexpected findings. A significant association between ED and PAD was observed in subjects without hypertension, diabetes, cardiovascular disease (CVD), and high cholesterol. In contrast, this association was not significant in subgroups with these conditions. This seemingly paradoxical phenomenon may be attributed to several factors: Firstly, individuals with these conditions may have already adopted more proactive health management strategies, such as lifestyle improvements or pharmacological interventions, which could influence the relationship between ED and PAD. Secondly, these conditions themselves might mask the potential association between ED and PAD. Lastly, this disparity may reflect complex pathological mechanisms or unidentified confounding factors. However, these explanations remain speculative and require validation through further research. Future studies should focus on exploring the potential mechanisms underlying the association between ED and PAD in these subgroups, as well as the impact of various interventions on this association. We reviewed all previous NHANES studies on PAD and found similar results in some subgroup analyses: there was a significant association between dietary magnesium intake and PAD in diabetes-negative and hypertension-negative subgroups (29); there was a significant association between the dietary inflammatory index and PAD in diabetes-negative, hypertension-negative, and CVD-subgroups (15).

Smoking has long been acknowledged as a major avoidable risk factor for PAD. Our findings demonstrated that former smoking habits may lead to a persistent increase in PAD risk, consistent with previous research (30). No significant relationships were observed between PAD and alcohol consumption. The relationship between drinking alcohol and CVD remains controversial (31). Epidemiological studies have revealed a U or J-shaped association between alcohol intake and cardiovascular conditions, such as heart attacks and strokes. This indicates an increased likelihood of CVD in individuals who do not drink alcohol and heavy drinkers, highlighting a beneficial impact linked to moderate alcohol intake (32, 33). Moreover, the current study confirmed the association between ED and PAD in physically inactive individuals, which aligns with previous reports. Kulinski found that exercise time was inversely associated with a low ABI (34).

Interestingly, our study uncovered an association between ED and PAD only in the overweight group. Obesity is a major public health issue and is strongly associated with atherosclerosis and heart problems. Despite frequent co-occurrence, the relationship between obesity and PAD remains controversial. Some studies suggest an unexpected protective effect of obesity, termed “obesity paradox” (35). A recent study discovered that obesity was primarily linked to PAD in women, while men showed only a minor correlation between higher BMI and PAD (36). Possible explanations include genetic factors, adipose tissue dysfunction, and differences in body fat distribution.

The present study possesses several strengths. Firstly, this study relies on NHANES data, offering the advantage of a large sample size. Secondly, this study allows for the adjustment of crucial PAD risk factors. Finally, we conducted subgroup analyses and adjusted for pertinent covariates, thereby augmenting the robustness of the study. Nevertheless, this study has some limitations. First, the cross-sectional study design does not allow for the determination of causality. Therefore, comprehensive longitudinal studies are warranted to validate our findings. Second, the lack of ABI data for participants aged <40 years old limited our ability to analyze this association across a broader age spectrum, despite PAD predominantly affecting older populations. Third, ED evaluation relied on self-reported assessment surveys, introducing inherent biases. Finally, the various impacts originating from eating disorders and PAD are complex. Although our adjustment model incorporated relevant covariates, fully mitigating the effects of other potential covariates remains challenging.

## 5 Conclusion

In summary, this large cross-sectional study suggests a significant association between ED and PAD in the US population ≥40 years old. ED may be an independent predictor of PAD and thus it should be considered in the treatment of patients with ED.

## Data Availability

The original contributions presented in the study are included in the article/supplementary material. Further inquiries can be directed to the corresponding author.
